# Supplementary material for: Enhanced antitumoral activity of TLR7 agonists via activation of human endogenous retroviruses by HDAC inhibitors
Source: Commun Biol. 2021 Mar 3;4:276. doi: 10.1038/s42003-021-01800-3 (PMC7930250; doi:10.1038/s42003-021-01800-3)
Supplement: Supplementary file 1 — Supplementary Information [file 42003_2021_1800_MOESM1_ESM.pdf]

Supplementary Table 1: HERV IDs, sequences and polarities of primers and probes

	Fw. Primer (5' - 3')	Probe (5' - 3')	Rev. Primer (5' - 3')
18 S (NR_003286)	GGACATCTAAGGGCATCACAG	TGCTCAATCTCGGGTGGCTGAA	GAGACTCTGGCATGCTAACTAG
HERV-IDDM-K 1,2 (AF012337.1)	CACCAGTCACATGGATGGATAA	AATGATAGTGTATGGGTACCTGGCCC	CCCTTCTTCCTCAGGTTTGG
HERV-W_{env} (AF072506.2)	GTGCCTGGGTTTGTTCTAATTG	AAGCCGTGGGTCACAGAAGAGAAC	ATCTTGGGCCATGTGGTAAG
HERV-W Protease (FN806835.1)	TGAAGACTGGGCAACAGAAG	TTGAACAGGACGGGCATTCTTTGC	GGTACTTCCTTTGGTAGGGAAAG
HERV-$K_{seminoma}$ (sem.) (72391228)	GTAGAGAGCCACCTGACTTATG	ACATTGGGCTAGTCAATGTCGTTCT	TGCTCGTTTCCCGACAAT
ERV-MER-34-1 (XR_941039.1)	CGAGCCTTCTTCTTGGTCTT	TTTATCCCTCGGAGTTGCACTGGC	CCGAAACCCGCTACAAAGTA
ERV-V_1 (NM_152473.2)	GACACCAGTGAGTCAAGTTAGG	CCACCATCCATCCCATGAGCTTCT	GTGACAGGAAGAACACCTCTG
ERV-V_2 (NM_001191055.1)	CCAAGAAGACCAAGAACCAGAG	CCTGGTTACCTCGCCTGGTTATCC	CTGGAGATGCCAGAAATGTTG
HERV-3-1 (NM_001007253.3)	GTAGTCTTCACTGCTCTGTGTC	CTTCTGTGGCCTCGTTACCTGCA	CTTAGCTGTGGATCTCCCAATAC
HERV-FRD_1 (NM_207582.2)	AAACACCAGGGACAGCTTATC	TCGCCCAGAGAATGGACAAGCAT	GCCTCATCAGTCCTTTCAGATT
HERV WE_1 (NM_001130925)	GAGTTCAAGATCAGGCAAGAGA	AAGAAGTAATCTCCCAACTCACCCGG	GTACGGAGGGTTTCATGTAGTT
HERV-C (AB266802.1)	GCTACCTTGCTCCCAGTATTAG	TCCCTGTCGAGCATGACTGTGTAGA	CATCCACGTATAGTTCCCAGTC
HERV-T_{env} (AB120695.1)	CCAGAAGGACGACAACTCATC	TGAGTTACATCCCAGGTTGCACCA	GCTATGCTAAGACCAGCCAATA
HERV-S_{pol} (AB162179.1)	AGGAGCTGCAAATAGTGAGTG	TCAGAAAGCCTGATGGGACTTGGC	TGCTTCATTCAGTTCCCGATAA
HERV-F_{pol} (AB120695.1)	GATGCTAACATTCAGCCCAAATAC	TCTACATCTCCTCTCCTCCCAAGCA	GGGAAGGAGGAGGAGAACTTA
HERV-L (EF141078.1)	GACTAGTAGGGTGAGGGCTATTA	AAGCCATTTGAGCTGCCTCTACCT	CACCAGGGATGCAGTATTGT
HERV-K_{env} (DQ069916.1)	AGTCTTTCTACCCTTGGGAATG	TGGATGTTCAGGACCAGAAACAGGAC	GCCACAGTAAGCCTCCATAA
HERV-K_{prt} (DQ159119.1)	GCGAAATTCAGCTGGTGATTAG	CGATCGTATTGCGCAGCTGCTG	GCTGTTGCCGCCTTTAATATAC
HERV-K_{pol} (Y10391.1)	CACCAGTCACATGGATGGATAA	CAGGGCAGCGATCATCTATGGGTC	CCCTTCTTCCTCAGGTTTGG
HERV-K_{gag} (DQ157733.1)	GGCCAGTATTGATAGGCTACTG	CACAAGTCCTCTTCCAGCAGGTCA	CCATCAGAGTCTAAACCACGAG
HERV-K_{ISD} (DQ360584.1)	CAGTCAGTAAACTTTGTTAATGATTGGC	TGATCTTAGACAAACTGTCATTTGGATGGG	TGTTCTAAGCTCATGAGTCTGTCT
HERV-K_{LTR} (AB047320.1)	AAATATGGCCTCGTGGGAAG	CCCATAAAGGGTCTGTAACTGAGGAGGA	CCTCTTGTCTCAACTGCAAGA
HERV-P_{env} (AB240043.1)	AGGCCTCTGAGGTAGTAAAGA	AAAGTGCCTCCAAAGTGAAATGGCC	GTGATGGCTGCTTCAAATGAG
HERV-P_{gag} (AB240024.1)	CCAGTGTTATCCAAAGGCTTCTA	TGCCAGCAACAGGATCTCCAACTT	GGAGATATGGTGGTTGCAGATAG
HERV-P_{pol} (AB240030.1)	CTCTCAATACCTGTCTGCCTTC	TGCCTCTGACCAAACCATCCAGTT	AATGCCTGTCCAAACAAATGAA
HERV-P G (AF081551.1)	CACCCAATCGGAGGTATCTTG	TGGTCAGGACATTCCAAGGCAACA	CCACGGAGGTCATGAAGTATT
HERV-E (AB259286.1)	GATGGCTTAATGGTGCCAATG	TACCTGATCAATGGGCAGGCAGTT	ACGGCTCCAGCTAGACTATA
HERV-FC_1 (AJ507128.1)	GCCTCATCAGTCCTTTCAGATT	TCGCCCAGAGAATGGACAAGCAT	AAACACCAGGGACAGCTTATC
HERV-$HC2_{pol}$ (AB167348.1)	GTGAATCCTTCCTTCTGGAACT	CAGGGAGCACACTCCAATCCAGTT	CCAACAGGTATGAGACACCATC
HERV-H_{env} (AH007767.2)	CAGTATTGATAGAGGGTCTGTCTG	CCGAAGCCCAACTACACACATCACT	GGTAATCTTTCACCTTCTCGATGT
TLR 7 (NM_016562.3)	GACTGCACAGACAAGCATTTG	CCTGGAGGTATTCCCACGAACAC	GGAGATGTCTGGTATGTGGTTAAT

Supplementary Table 2: Screening profile for HERV-specific RNA expression in SKOV3[WT] ovarian carcinoma cell line using qPCR

HERVs	ΔC_t		
HERV $HC2_{pol}$	25,99	±	0,57
HERV $V_{3.1}$	19,32	±	0,41
HERV C	26,20	±	0,36
HERV E_{env}	20,10	±	0,51
HERV F_{pol}	19,69	±	0,37
HERV Fc_1	29,68	±	0,57
HERV FRD_1	21,71	±	0,60
HERV H_{env}	15,87	±	0,56
HERV $IDDMK_{1,2}$	18,68	±	0,41
HERV K_{ISD}	17,66	±	0,23
HERV K_{env}	18,49	±	0,45
HERV K_{gag}	19,47	±	0,58
HERV K_{LTR}	19,91	±	0,60
HERV K_{pol}	18,16	±	0,48
HERV L	28,42	±	1,52
HERV MER34.1	27,96	±	0,43
HERV P_{env}	25,26	±	0,48
HERV P_{gag}	25,33	±	0,31
HERV P_{pol}	26,05	±	0,31
HERV $P_{4.6}$	32,52	±	1,25
HERV S_{pol}	32,00	±	0,73
HERV T	28,36	±	0,87
HERV V_1	**22,95**	**±**	**1,89**
HERV V_2	**23,11**	**±**	**2,07**
HERV W_{env}	22,12	±	0,53
HERV W_{prt}	20,48	±	0,72
HERV WE_1	22,12	±	0,62
HERV $K_{seminoma}$	18,90	±	0,41

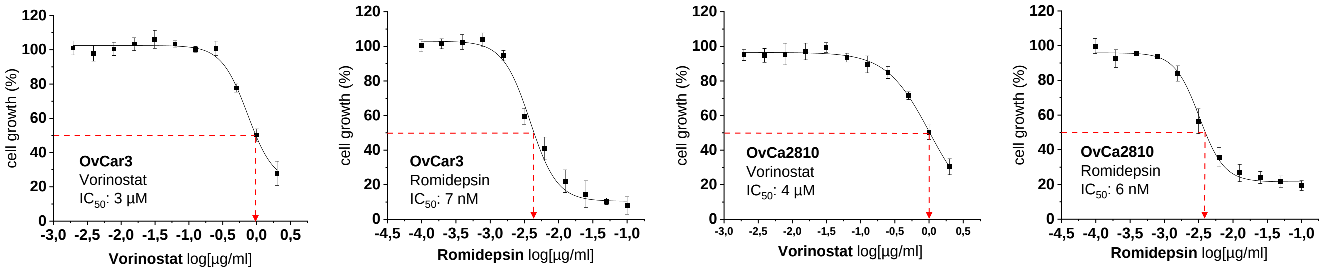

Supplementary Figure 1: The cytotoxicity of the HDACis vorinostat and romidepsin in OvCar3^{WT} and OvCa2810 primary ovarian carcinoma cells was determined using the MTT proliferation assay after 72h of drug exposure. Results are representative of n≥3 experiments.

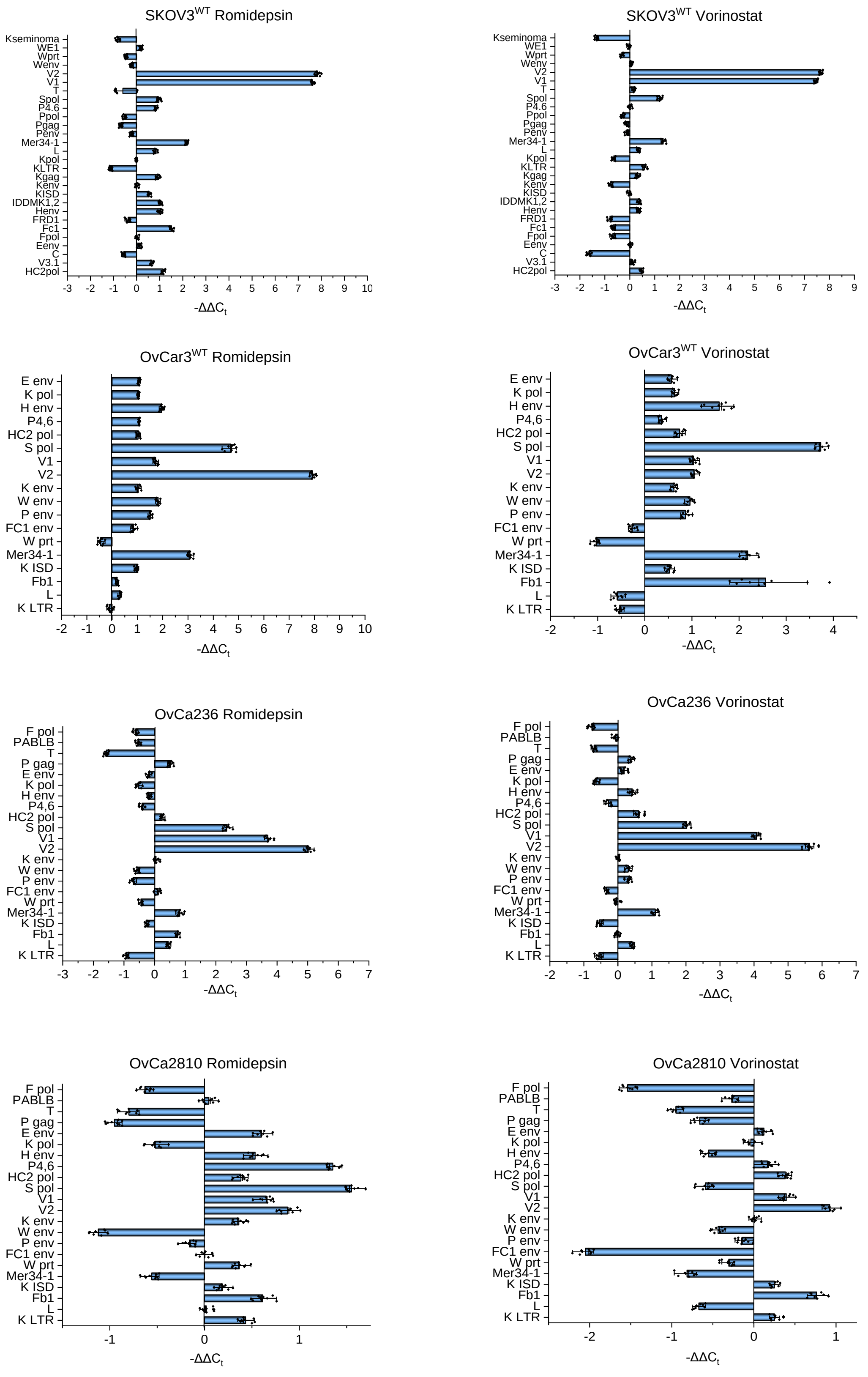

Supplementary Figure 2: Overexpression of several HERVs was observed after HDACi treatment in different ovarian carcinoma cells as determined by qPCR. Results are representative of n≥3 experiments.

a

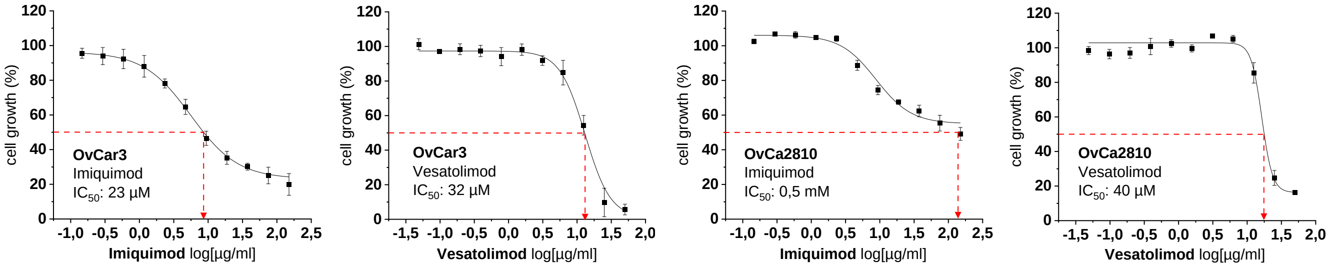

b

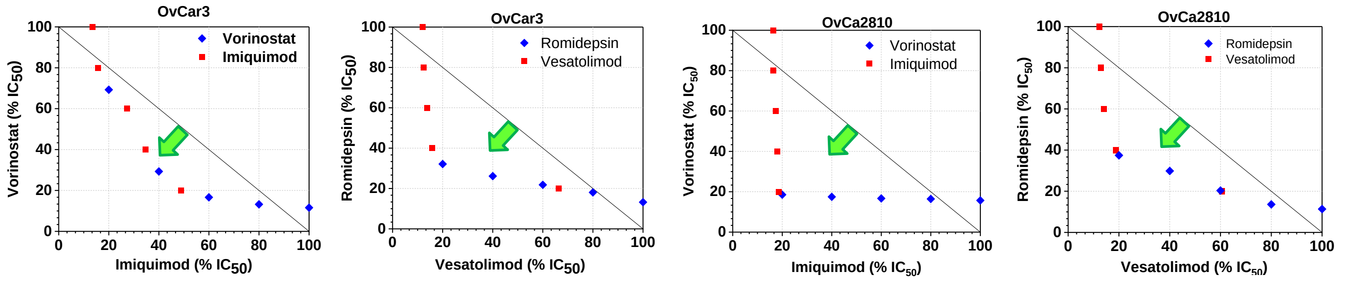

c

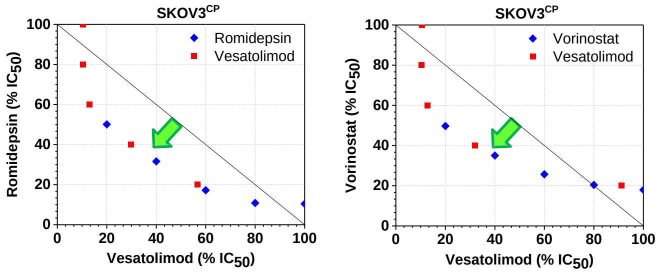

Supplementary Figure 3: **a**. The cytotoxicity of the TLR7as imiquimod and vesatolimod in OvCar3^{WT} and OvCa2810 primary ovarian carcinoma cells was determined using the MTT proliferation assay after 72h of drug exposure. Results are representative of n≥3 experiments. **b**. Synergistic cytotoxic effect was observed by interaction of HDACis and TLR7as using isobolographic methods in OvCar3^{WT} and OvCa2810 primary ovarian carcinoma cells isolated from ascites. Results are representative of n=5 experiments. **c**. Synergistic cytotoxic effect was observed by interaction of HDACis and TLR7as using isobolographic methods in SKOV3^{CP} cells. Results are representative of n=5 experiments.

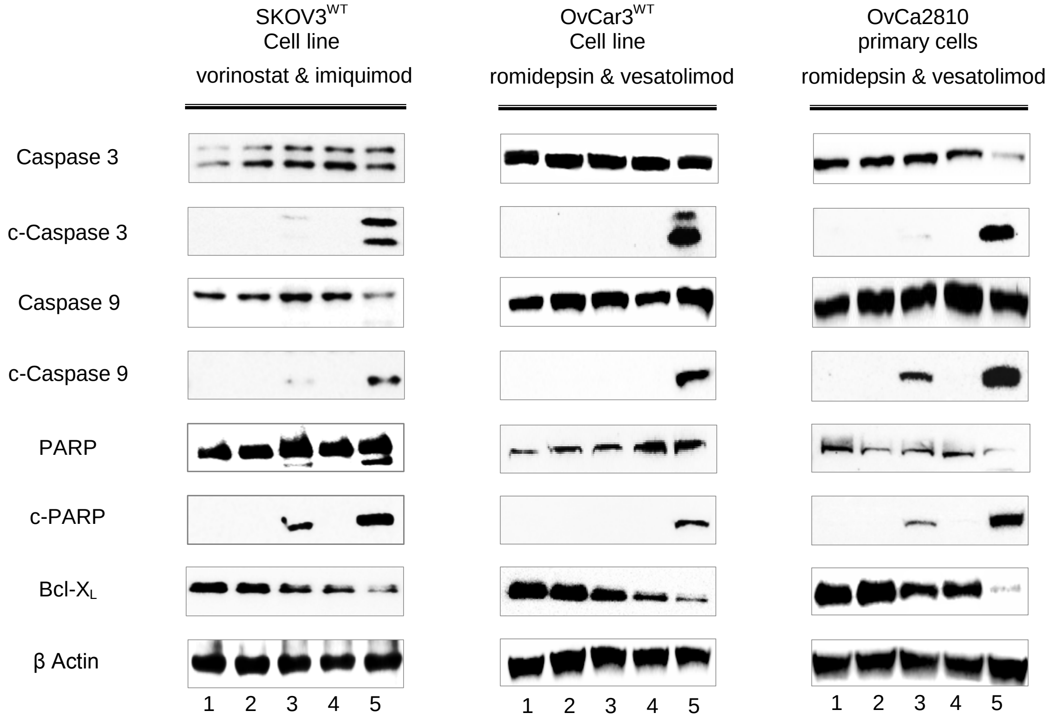

Lanes: **1**: untreated cells; **2**: DMSO control; **3**, HDACis; **4**, TLR7as; **5**, HDACi+TLR7a combination

Supplementary Figure 4: After the incubation of SKOV3[WT], OvCar3[WT] and OvCa2810 cells with 1-fold their respective IC_{50}-values for HDACis, TLR7as and their combinations for 24h, apoptosis mediators were analyzed by Western blots revealing the cleavage of central apoptosis pathway mediators such as caspase 3, as well as caspase 9, an element which is determinant for intrinsic activation of cell death. In congruence, the specific cleavage of PARP in the combination exposed the specificity of the interaction of both substance groups. Moreover, an anti-apoptotic protein Bcl-xL was down regulated only in this combinatorial treatment. Results are representative of n≥3 experiments.

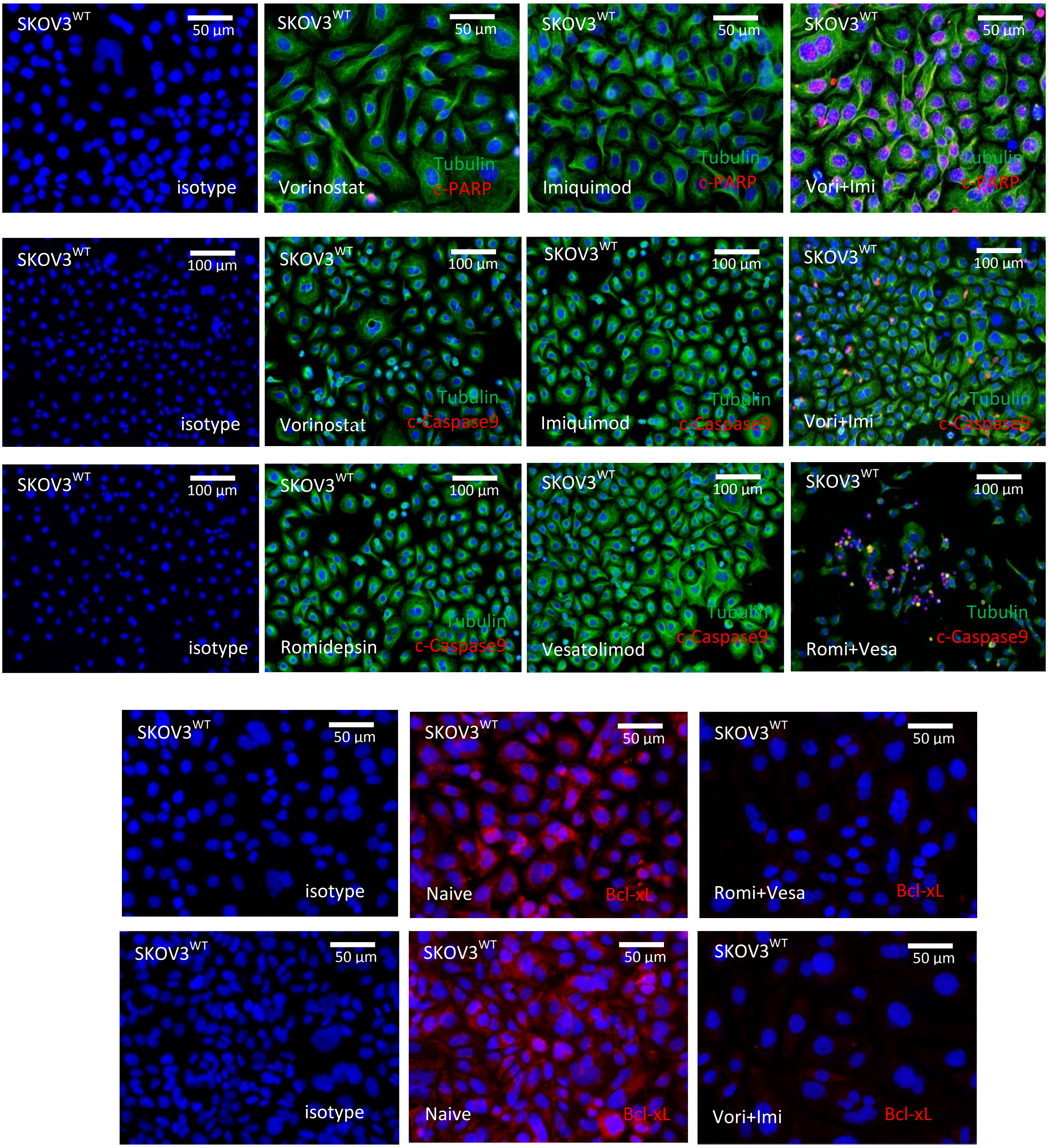

Supplementary Figure 5: ICC analysis of cleaved PARP, cleaved caspase 9 and Bcl-xL in SKOV3^{WT} cells after incubation with HDACis, TLR7/8as and their combination mirrored the effects observed in Western blots. Our protein of interest in red, DAPI in blue, beta tubulin for cellular structures in green. c-PARP image panel magnification 40x, c-Caspase 9 and Bcl-xL image panels magnification 20x. Results are representative of n≥3 experiments.

a

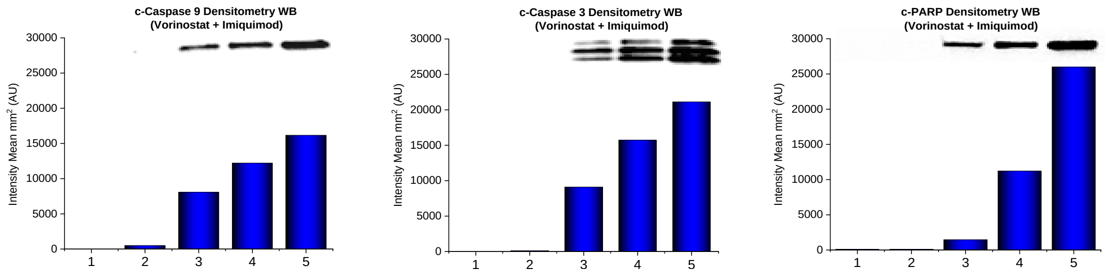

Lanes: 1: control, 2: 0.125x, 3: 0.25x, 4: 0.50x and 5: 1.0x of the IC_{50} in SKOV3^{WT}

b

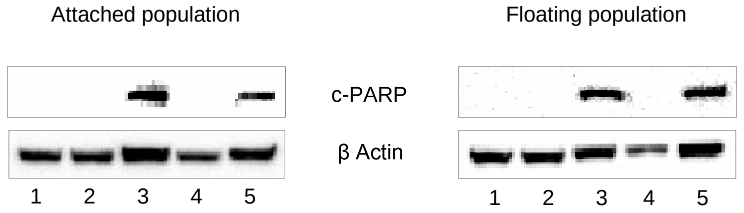

Lanes 1: Naive, 2: DMSO 48h, 3: Romidepsin + Vesatolimod 48h; 4: DMSO 72h and 5: Romidepsin + Vesatolimod 72h

c

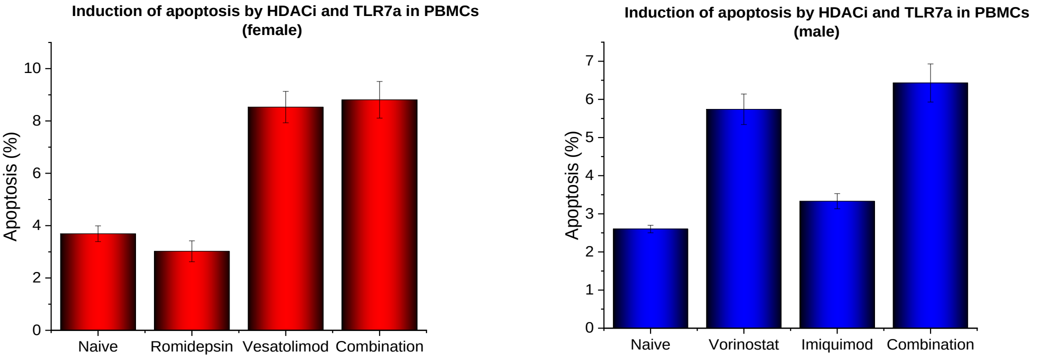

Supplementary Figure 6: **a**. Dose-dependent expression of cleaved caspases 3, 9 as well as PARP was observed in SKOV3^{WT} cells treated with vorinostat and imiquimod combination. Results are representative of n≥3 experiments. **b**. SKOV3^{WT} cells were treated with a combination of romidepsin and vesatolimod with 1 fold IC_{50} concentration for a time period of 48h and 72h. In both time frames, control cells were alive and remained attached to the culture flask. Cells treated with DMSO also remained attached to the cell culture flask. After the combinatorial treatment, some cells float while some cells were still attached to the culture dish. These two populations of cells were separated and analyzed for the expression of cleaved-PARP by western blots. Results are representative of n=3 experiments. **c**. Apoptotic events detected after the simultaneous incubation of HDACis and TLR7as in PBMCs from a total of 10 healthy volunteers. Freshly isolated PBMCs were exposed to IC_{50} of drugs, either as single treatment or combined exposure for 24h. Vesatolimod and vorinostat significantly induced apoptosis in leukocytes. The combination of both drug classes apparently does not induce more apoptosis than each single drug. Apoptosis was measured by Annexin V method. Graphs are representative of a total of 3 experiments.

a

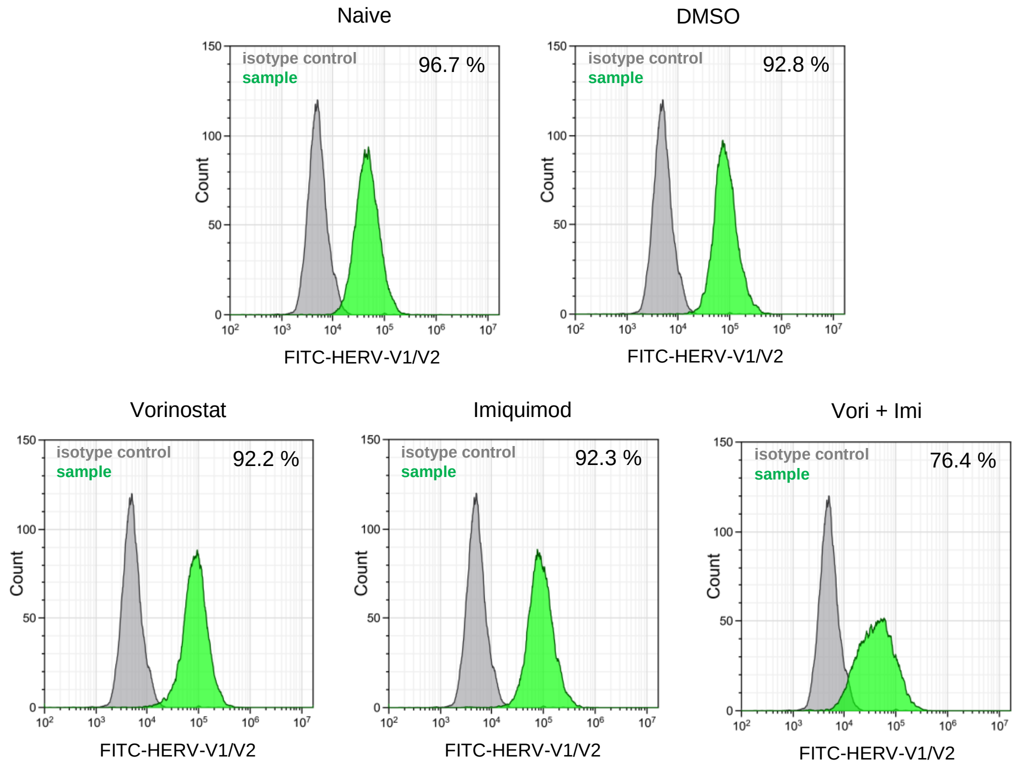

b

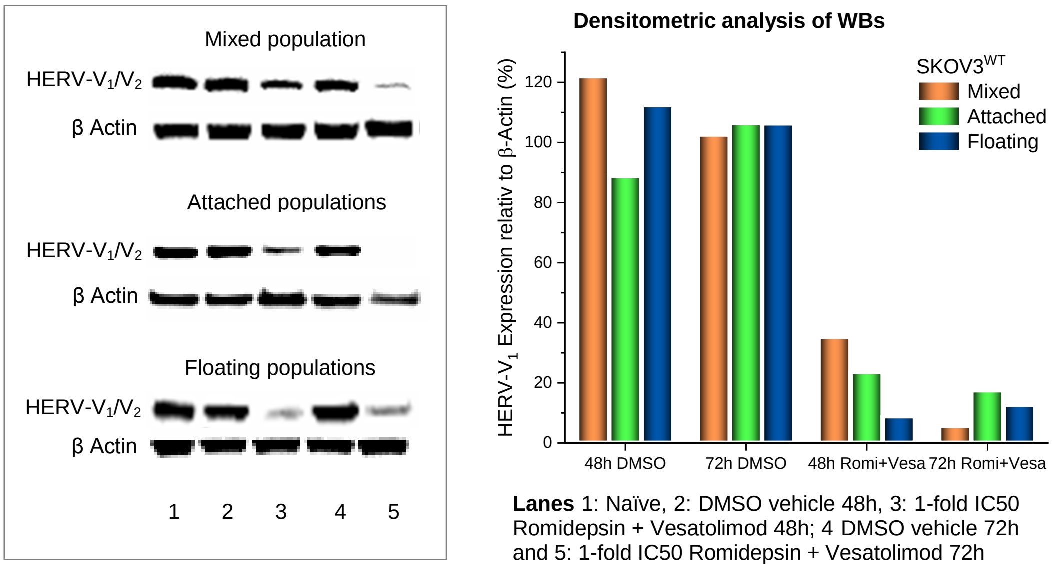

Supplementary Figure 7: **a**. Vorinostat and imiquimod alone do not stimulate HERV V_1/V_2 protein production; instead, a reduction after simultaneous incubation with both substances is observed by cytometric analysis in $SKOV3^{WT}$. Results are representative of n≥3 experiments. **b**. Reduction of HERV-V_1/V_2 after the simultaneous exposure to romidepsin and vesatolimod in $SKOV3^{WT}$ cells after 48h and 72h. Results are representative of n≥3 experiments.

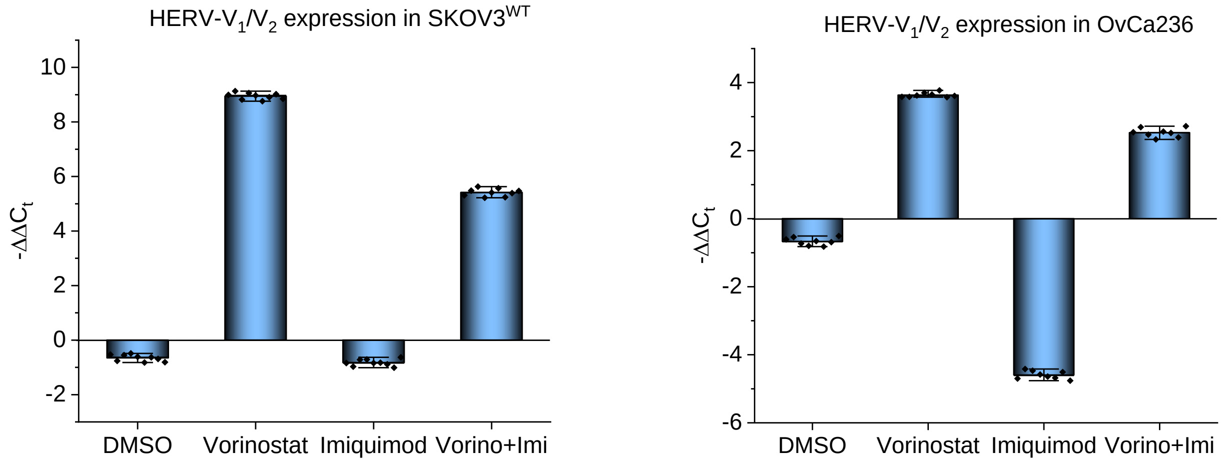

Supplementary Figure 8: Influence of vorinostat and imiquimod on the expression of HERV V_1/V_2 transcripts in SKOV3^{WT} and OvCa236 cells. The HDACi vorinostat acts as reactivator of HERV V_1/V_2 transcription, as detected by qPCR. Contrarily, imiquimod represses transcription if co-administered at 1× IC_{50} for 24 h. Results are representative of n=3 experiments.

a

```
121                                      atggtgtttc caatgtggac actgaagaga
181  caaattctta tcctttttaa cataatccta atttccaaac tccttggggc tagatggttt
                                                        Probe 276
241  cctaaaactc tgccctgtga tgtcactctg gatgttccaa agaaccatgt gatcgtggac
                                                  Probe 391
301  tgcacagaca agcatttgac agaaattcct ggaggtattc ccacgaacac cacgaacctc
                                    3-gga cctccataag ggtgcttgtg-5(- Strand)
361  accctcacca ttaaccacat accagacatc tccccagcgt cctttcacag //////////
1021 gaattaaaag ttttacgtct acacagtaac tctcttcagc atgtgcc
```

b

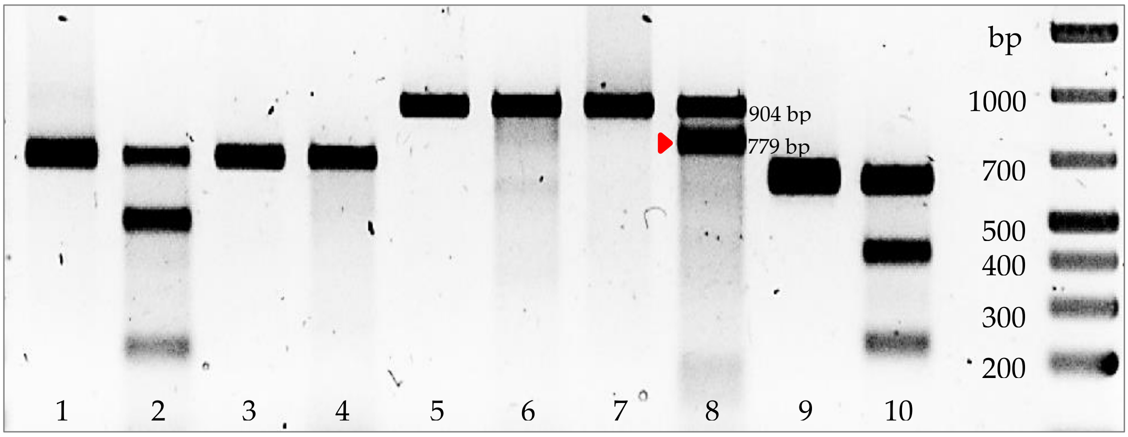

Lanes: **1**: HPRT positive control Ø T7 digestion; **2**: HPRT positive control T7 digestion (full length 669, cut 1: 198 bp, cut 2: 471); **3**: Negative control Ø T7 digestion (scramble gRNA); **4**: Negative control T7 digested (scramble gRNA); **5**: Control only electric pulse (4D Nucleofector, code FE132) Ø T7 digestion; **6**: Control only electric pulse (4D Nucleofector, code FE132) T7 digested; **7**: TLR7 KO Ø T7 digestion; **8**: TLR7 KO T7 digested (red arrow); **9**: NEB kit control for T7 enzyme, undigested; **10**: NEB kit control for T7 enzyme, T7 digestion

c

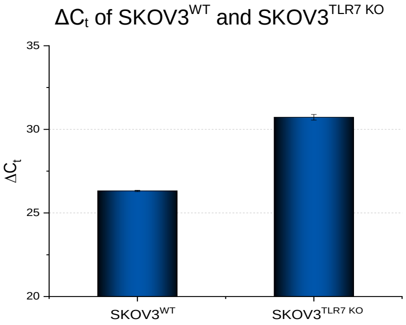

Supplementary Figure 9: **a**. *TLR7* gene and design of gRNAs, primers and probes for analysis of gene ablation. NCBI Reference Sequence: NM_016562.4. Turquoise: Primers for T7 (904 bp); Yellow: TLR7 gRNA1 and TLR7 gRNA2; Grey: qPCR primers for gRNA1; Red: Probe 276 for TLR7 gRNA1 and Probe 391 for TLR7 gRNA2; Green: qPCR primers for gRNA2. The forward slashes represent the omitted sequence in this sector. **b**. Results of the CRISPR/Cas9 T7 digestion products in SKOV3$^{TLR7\text{-}KO}$ cells separated by 2% agarose gel. **c**. Successful knock out of TLR7 gene as assessed by qPCR in CRISPR/Cas9-modified SKOV3 cells.

a

```
 781 caaaaaaata aactgccctt tgatggaagt cctaagataa cttattcaac cccccctgtg
 841 gcaaacctct acacttgcat taataacatc caacatacgg gagaatgtgc tgtgggactt
 901 ttgggaccac gggggatagg tgtgaccatt tataacacca cccaacccag //////////
1201 gtgatcagta aatcctgttg catttatgtc aataacagtg gggcgataga ggaggatata
                                 Probe 280
1261 aaaaagatct atgatgaggt tacgtggctc cataactttg gaaaaggtga ttcagcaggg
1321 tccatttggg aggctgtgaa gtctgccctc ccctccctca catggtttgt ccctttactg
1381 ggaccagctg cacttaatag cctgctttct cctctttggc ccttgtctct //////////
1801 agcccgctga tggcctcctt ggaaacgctg cacgctgagt cagcaaaagg aggagcgtgg
```

b

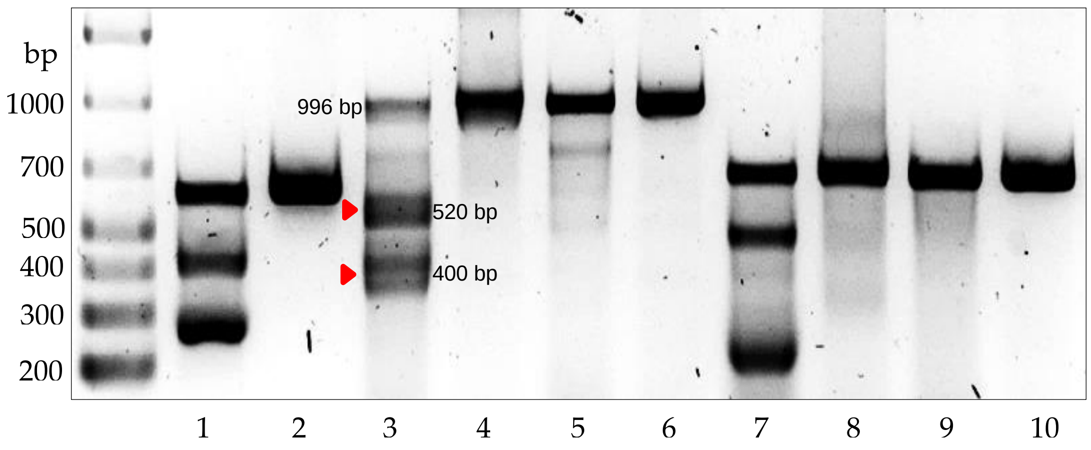

Lanes: **1**: NEB kit control for T7 enzyme, T7 digestion; **2**: NEB kit control for T7 enzyme, undigested; **3**: KO HERV-V_1 T7 digested (red arrows); **4**: KO HERV-V_1 Ø T7 digestion; **5**: Control only electric pulse (4D Nucleofector, code FE132) T7 digested; **6**: Control only electric pulse (4D Nucleofector, code FE132) Ø T7 digestion; **7**: HPRT positive control Ø T7 digestion; **8**: HPRT positive control T7 digestion (full length 669, cut 1: 198 bp, cut 2: 471); **9**: Negative control Ø T7 digestion (scramble gRNA); **10**: Negative control T7 digested (scramble gRNA)

c

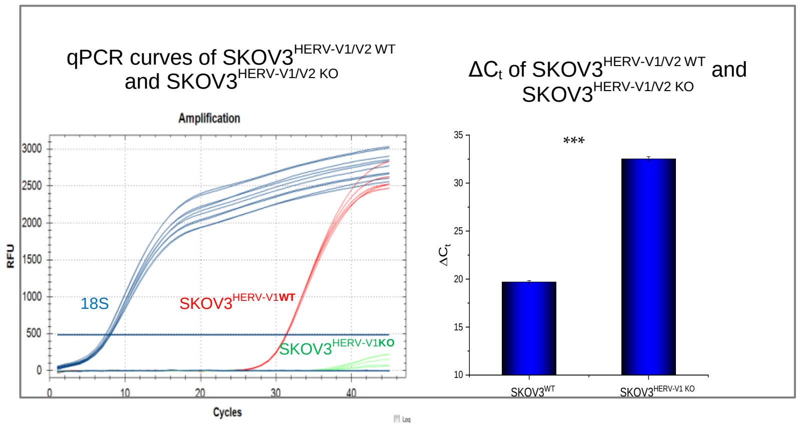

d

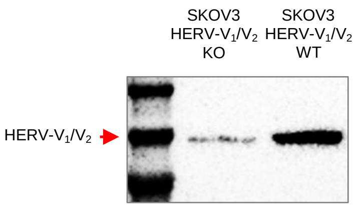

Supplementary Figure 10: **a**. *HERV-V1* gene and design of gRNAs, primers and probes for analysis of gene ablation. NCBI Reference Sequence: NM_152473.2. Turquoise: Primers for T7 (996 bp); Yellow: HERV V_1 gRNA1 and gRNA2 (black underline indicates overlapping sequences); Grey: qPCR primer for gRNA; Red: Probe 280 for HERV V_1 gRNA1 and 2. The forward slashes represent the omitted sequences in this sector. **b**. Results of the CRISPR/Cas9 T7 digestion products in SKOV3$^{V1/V2\text{-}KO}$ cells separated by 2% agarose gel. **c**. Successful knock out of HERV-V1 gene as assessed by qPCR in CRISPR/Cas9-modified SKOV3 cells. **d**. Successful knock out of HERV-V1 gene as assessed by Western Blot in CRISPR/Cas9-modified SKOV3 cells.

a

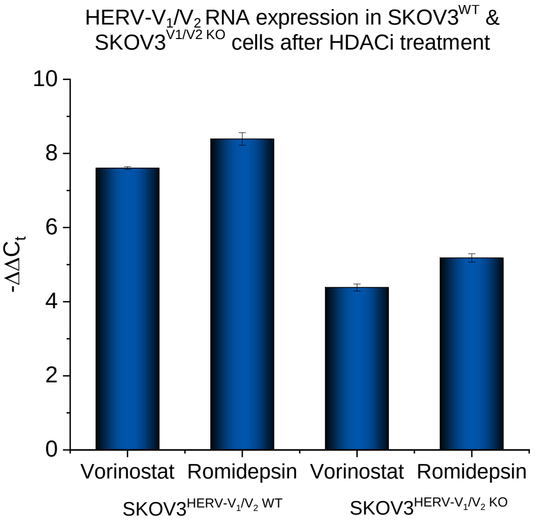

b

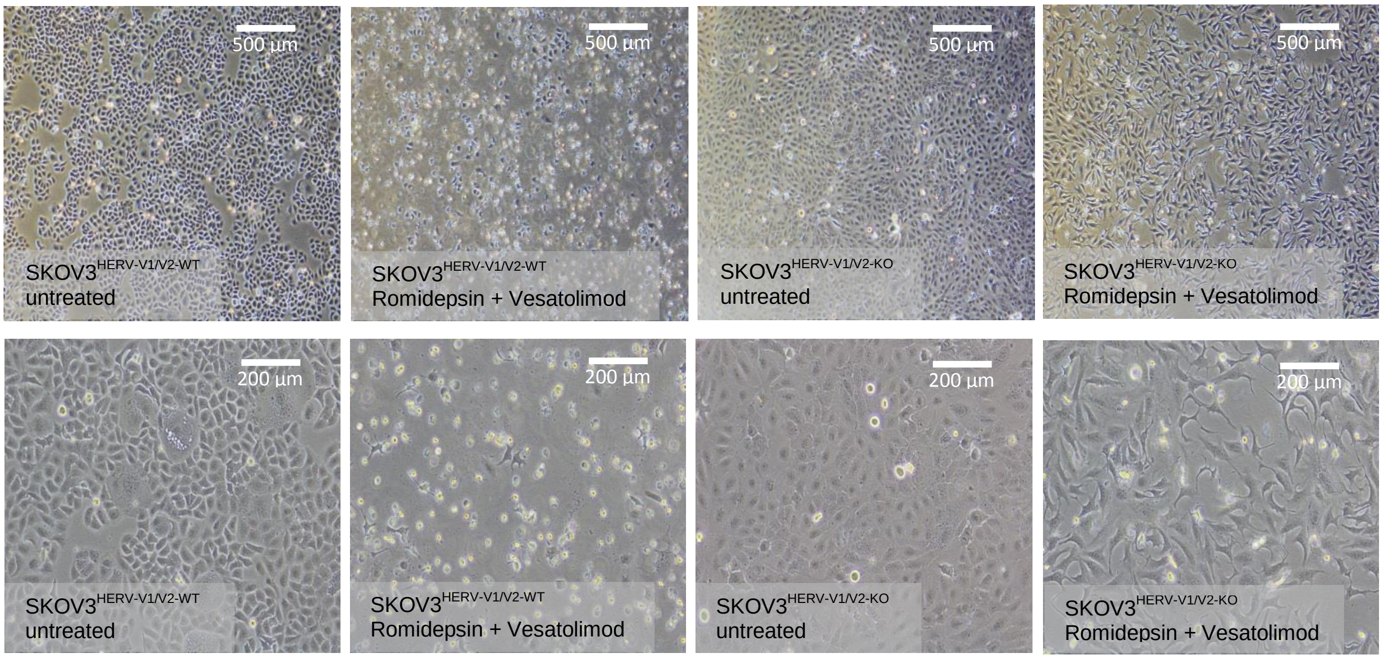

Supplementary Figure 11: **a**. Alterations in HERV-V_1/V_2 RNA transcription in the SKOV3$^{V1/V2\ KO}$ cells after HDACi treatment compared to SKOV3^{WT} cells as assessed by qPCR. In these KO cells, HERV-V_1/V_2 transcripts were less upregulated in SKOV3$^{V1/V2\ KO}$ cells compared to SKOV3^{WT} cells after HDACi treatment. This could be the result of the existence of non-ablated population. Results are representative of n=3 experiments. **b**. Microscopic studies revealed that there is clear apoptosis reduction in V_1/V_2 knocked out SKOV3 cells compared to their wild type when exposed to combination therapy. This confirms the role of HERV-V_1/V_2 as apoptosis mediators. Magnification 4x and 10x. Results are representative of n=5 experiments.

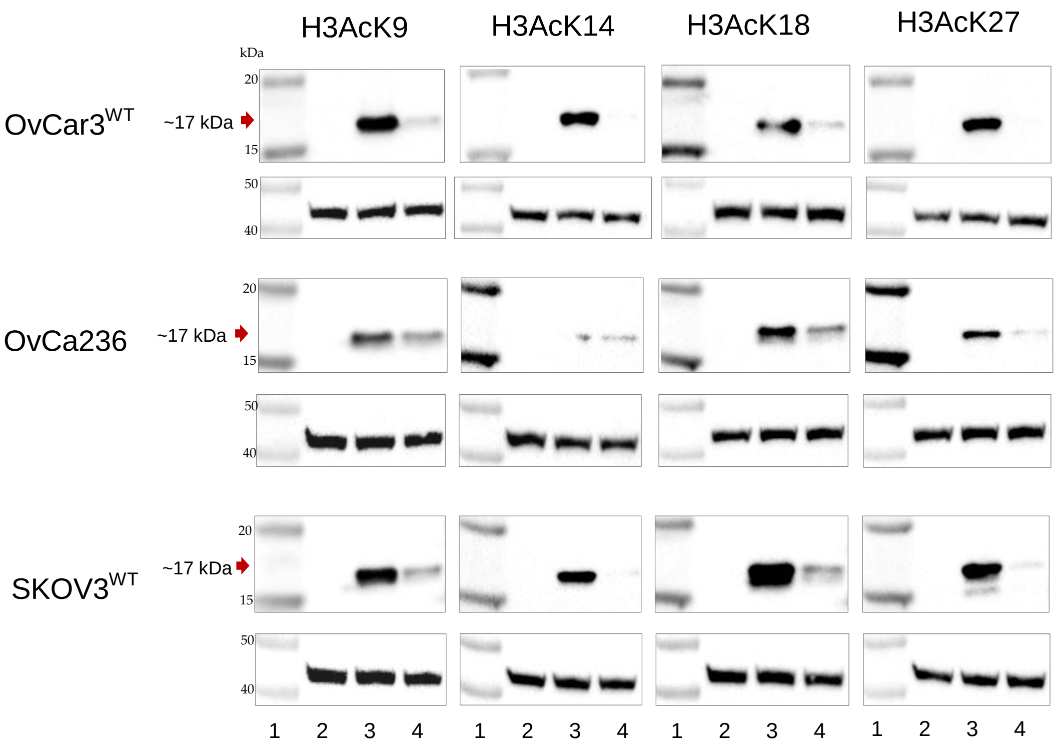

Lanes 1: Marker, 2: Untreated cells, 3: Romidepsin 4: Vorinostat

Supplementary Figure 12: Histone 3 acetylation pattern studies in OvCar3^{WT}, OvCa236 and SKOV3^{WT} cells after HDACi treatment. Overall, H3AcK9 seemed to be significant in all cell lines for both conditions. Results are representative of n≥3 experiments.

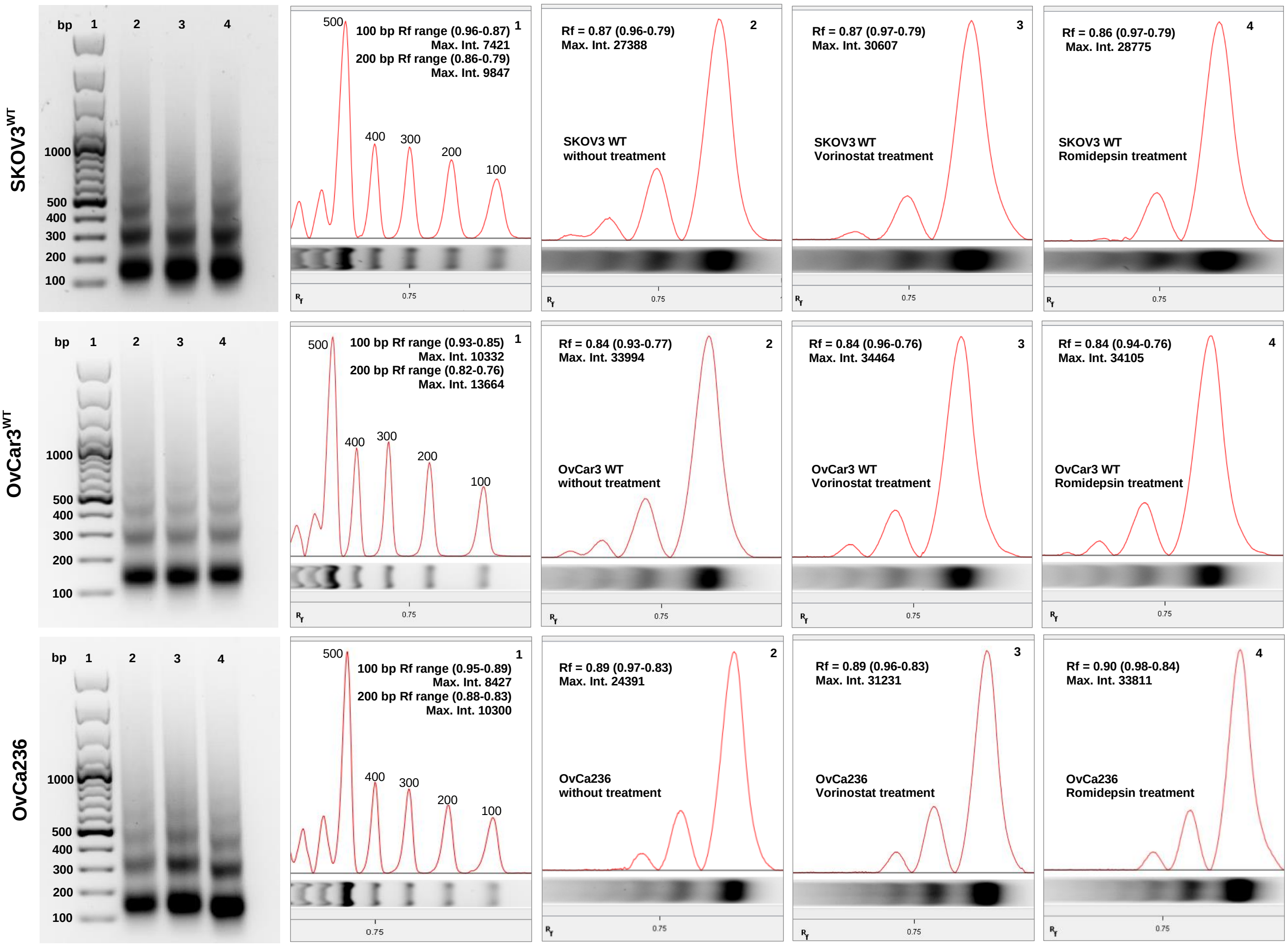

Supplementary Figure 13: Chromatin digestion analysis before immunoprecipitation. For each condition, 0.75 µg chromatin was loaded on to 1% agarose gel. Successful digestion was confirmed and most of the digested chromatin is 150 bp to 160 bp. The experiments were performed in SKOV3^{WT}, OvCar3^{WT} and OvCa236 cells. Lanes 1: marker, 2: untreated cells, 3: vorinostat treated cells, 4: romidepsin treated cells. Results are representative of n=3 experiments.

sample	peaks	HERV peaks	n° of HERV loci	diffpeaks	HERV diffpeaks	n° of HERV loci
Untreated	376.666	2.506	1.233 (37,6 %)	-	-	-
Vor-1	912.311 (x2,4)	6.220 (x2,5)	1.948 (59,4 %) (+715 loci)	7.661	129	29 (0,9 %)
Vor-2	913.199 (x2,4)	6.209 (x2,5)	1.974 (60,2 %) (+741 loci)	7.772	178	31 (0,9 %)
Rom-1	896.878 (x2,4)	5.695 (x2,5)	1.617 (49,3 %) (+384 loci)	12.314	298	55 (1,7 %)
Rom-2	884.242 (x2,4)	5.549 (x2,5)	1.597 (48,7 %) (+364 loci)	12.401	382	62 (1,9 %)

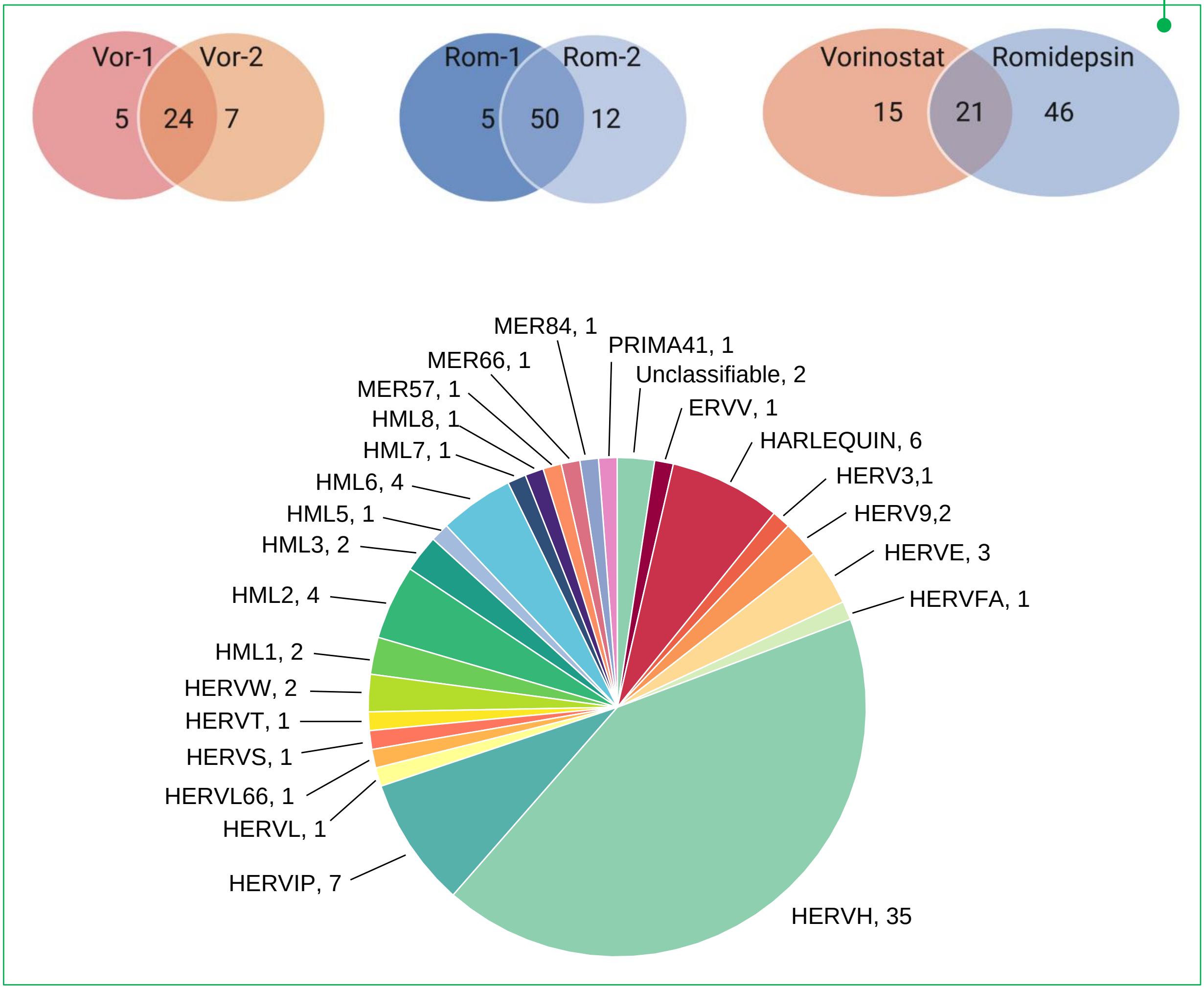

Supplementary Figure 14: Overview of HDAC inhibitors' effect on HERV loci based on CHIP-seq analysis. The number of peaks and differential peaks mapping at HERV coordinates are reported in the table (upper part) with the corresponding number of colocalised HERV loci. Percentages are referred to the total dataset considered, which includes about 3280 HERV integrations classified into 31 taxonomical groups. The graphs in the lower part of the figure show the number of HERV loci colocalised with diffpeaks for each treatment and the comparison among the different treatments. Their distribution in the different HERV groups is also reported with the group name followed by the number of HERV loci involved (the ERV-V group, including ERV-V2 locus, is highlighted in berry colour).

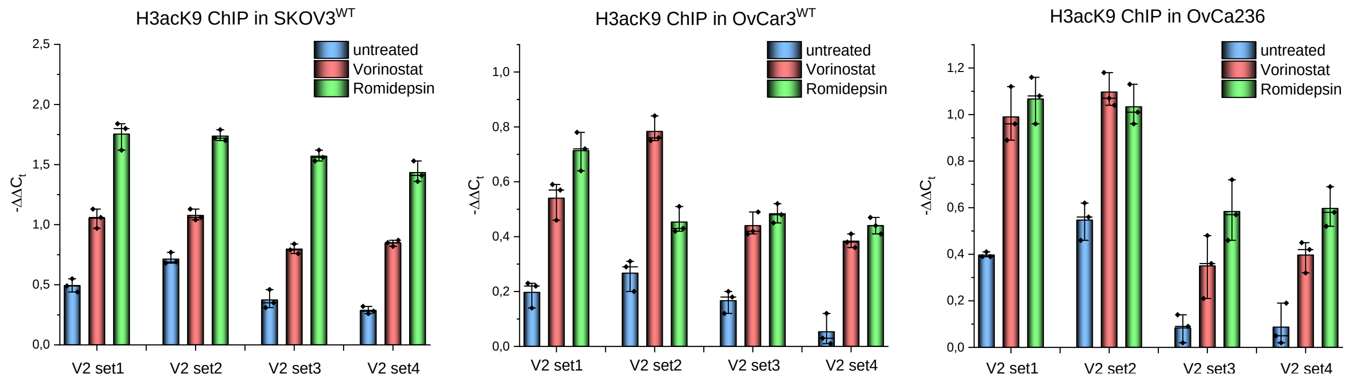

Supplementary Figure 15: ChIP-qPCR analysis performed in the cell lines SKOV3^{WT}, OvCAr3^{WT} and the primary ovarian carcinoma cell OvCa236. Two different sets of primers and probes were specifically designed for HERV-V_2. It is clear that the transcriptional expression of this HERV is dependent of the acetylation of histone 3 at lysine 9 (H3AcK9). These data are supportive of the ChIP-sequencing shown in the main document. Results are representative of n=3.

Supplementary Table 3: ERVV-2 sequences and polarities of primers and probes

ERVV-2	Fw. Primer (5‘ - 3‘)	Sonde (5‘ - 3‘)	Rev. Primer (5‘ - 3‘)
Set1	GACACCAGTGAGTCAAGTTAGG	CCAACCATCCATCCCATGAGCTTCT	GTGACAGGAAGAACACCTCTG
Set2	CCCTCCATGTGATTAGCTCTTT	AAACCGCTGTGCAGCTTGGG	AACTTGACTCACTGGTGTCC
Set3	GGAGATGTGCTTATAGGTGCTG	TCAGCTATCTGTCATTGGAGGCCC	AAGTGTGTCTCTTCTAGGATAAAGC
Set4	GTTTCCCGGAATCTTTGCC	CCCAGCACCTATAAGCACATCTCCC	GATATCAGCTATCTGTCATTGGAG

Supplementary Table 4: Inflammasome gene IDs, sequences and polarities of primers and probes

	Fw. Primer (5‘ - 3‘)	Probe (5‘ - 3‘)	Rev. Primer (5‘ - 3‘)
CXCR1 (NG_011814.1)	CAAGGTCAGGGCAAAGAGTAG	TGATGTCTACCTGCTGAACCTGGC	CTGAGCCTGCTGGGAAAC
CXCR2 (NG_052975.1)	AGGCTAGGTTCAGCAGGTA	AAACTCCCTCGTGATGCTGGTCAT	GGTCATTATCTATGCCCTGGTATT
IFI-16 (NM_001206567.2)	GTCTTGATGACCTTGATGTGACTA	CACCTGCTTTGAATTGGCACCGAA	TCAACTGTGAGGAAGGAGATAAAC
IFI-27 (NM_001130080.3)	CAATGGAGCCCAGGATGAA	AGTCACTGGGAGCAACTGGACTCT	CTCCATAGCAGCCAAGATGAT
IFI-44 (NM_006417.5)	AAGTCATCTGCAGCCCATATG	TGAGTGGGAGCTGGACCCTGTAAA	TTTCTGACATCTCGGTGGTTAG
IFN alpha (NM_024013.3)	AGGGCTGTATTTCTTCTCTGTC	ATGAATGCGGACTCCATCTTGGCT	ATGCAGGAGGAGAGGGT
IFN beta1 (NM_002176.4)	CCTTAGGATTTCCACTCTGACTATG	TGCATTACCTGAAGGCCAAGGAGT	GAGCAGTCTGCACCTGAAA
IFN gamma (NM_000619.3)	GGAAAGAGGAGAGTGACAGAA	ATGCAGAGCCAAATTGTCTCCTTT	TCATGTCTTCCTTGATGGTCTC
IL8 (NM_000584.4)	CCACTCTCAATCACTCTCAGTTC	AGACATACTCCAAACCTTTCCACCC	TTTGCCAAGGAGTGCTAAAGA
IRAK1 (NM_001569.4)	CGGGCAGGGTTGATGATAA	CAGCTCTGCATCATCGTCGTCAGA	GGACACGGCAGGAGAATC
IRAK4 (NM_001114182.2)	AGACATTGACTAGCAACAGAGT	TGATGCTGATTCCACTTCAGTTGAAGCT	GGATGAACACCGTCAACCT
IRF-5 (NG_012306.1)	GTCTTTGAGGTCTGGGTTTGA	CTCGACTGCTGCTGGAGATGTTCT	CCCGAGAGAAGAAGCTCATTAC
IRF-7 (NM_001572.5)	GATGTCGTCATAGAGGCTGTTG	TGCTGCTATCCAGGGAAGACACAC	TGCCGAGTGCACCTAGA
JAK1 (NM_002227.4)	GAGCTTGGTGTTCTCGTCATA	TGACAAAGAGGAGAGATACGGCATGC	GAGTACACAGCAGAGGAACTG
JAK2 (NM_004972.4)	CATCCATCTGGTCTTGGTAATCT	CGATCATCTGTCCTTGTTTGTCATTGCC	CCAGCGGAATTTATGCGTATG
JAK3 (NM_000215.4)	ACAGGTCCTCAGCCAAGT	CCAGCGCCTATCTTTCTCCTTTGGG	TTCATGCAGCCTCTTGTCC
MAGE A4 (NM_001011548.1)	GAACAAGGACTCTGCGTCA	AATGAGGGTTCCAGCAGCCAAGAA	CTTACCCACTACCATCAGCTTC
MAGE B1 (NM_002363.5)	GGACTTTCATCTTGCTGGTTTC	CCCACGCTTTCAATTCCTGTGGG	AAATATCTGGAGTACAAGCAGGT
MyD88 (NM_001172567.2)	GGAACTCTTTCTTCATTGCCTTG	CAGGTGCCCTCAGAAGCGACT	ATTTGCACTCAGCCTCTCTC
NF KB (NM_003998.4)	ACTGGTCAGAGACTCGGTAAA	TGGAGACATCCTTCCGCAAACTCA	CTCCACAAGGCAGCAAATAGA
Stat1 (NM_007315.4)	GCTGGCACAATTGGGTTTC	AGGACCCTCATTCGTTCTGGTGC	GCACCTGCAATTGAAAGAACA
Stat2 (NM_005419.4)	AGTTCCTCTGTCACACCTAGT	ACGTTCAGGTGGTTCAGGAAAGGG	GGCAGAGTCAGGGTTTGATT
Stat3 (NM_139276.2)	CCTCAGGGTCAAGTGTTTGAA	ATGGAAGAATCCAACAACGGCAGC	TCTCAGAGGATCCCGGAAAT
TMEM 173 (NM_198282.4)	GAGAAATCCGTGCGGAGAG	ACGATGTCCCAAGAGCCTGAGC	GGCAGCTTGAAGACCTCAG
TNF (NM_000594.4)	GATGCGGCTGATGGTGT	CCAGGTCCTCTTCAAGGGCCAA	GAGGGCCTGTACCTCATCTA
TRAF6 (NM_145803.3)	AGGGCTTCCAGATGCATAAA	CTTGCTTTCCAGCGACCCACAATC	CGAAGAGATAATGGATGCCAAA

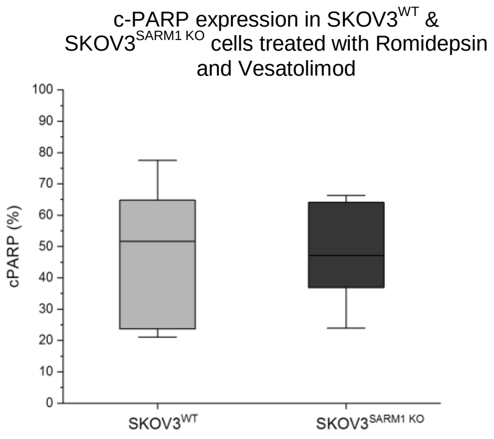

Supplementary Figure 16: SARM1 was ablated in SKOV3^{WT} cells using CRISPR/Cas9 and the expression of c-PARP was studied in KO cells after combination treatment by flow cytometry. There is no significant reduction in apoptosis in KO cells compared to WT, indicating that SARM1 is unlikely to be TLR7 adaptor in this cell line. Results are representative of n≥3 experiments.

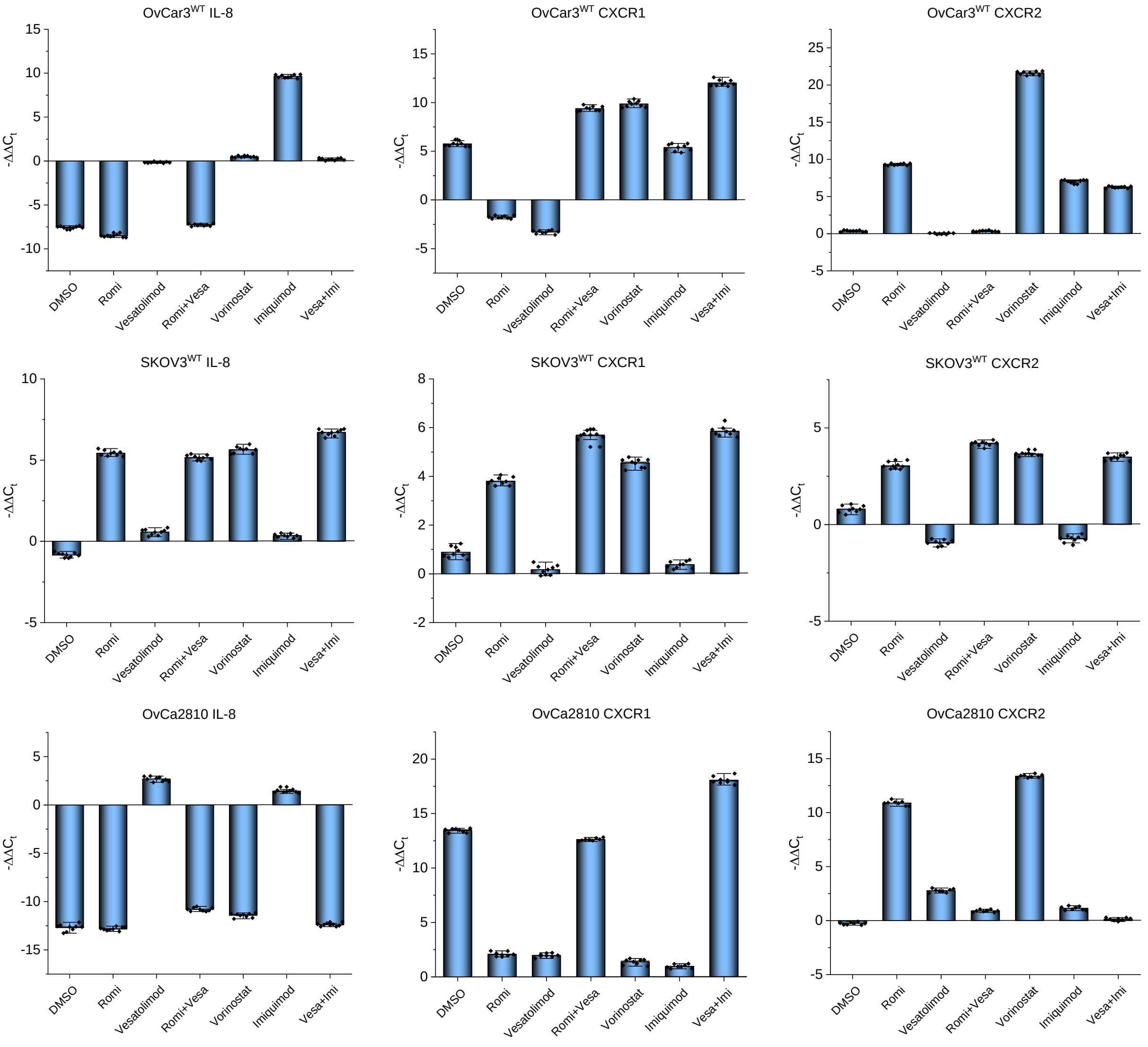

Supplementary Figure 17: IL-8 expression in ovarian carcinoma cells. Transcriptional status of IL8 in OvCar3^{WT} and SKOV3^{WT} and OvCa2810 ovarian carcinoma cells after the concurrent administration of HDACis and TLR7/8as. IL8 expression is driven by several transcription factors, among which NFκB is one of the most known TF for this IL. The co-administration of HDACis and TLR7/8as rendered in incongruent results, with tendency to show a downregulation of this interleukin. In the majority of primary ovarian cancer cells, there is a down-regulation after this interventional treatment. Oppositely, the SKOV3^{WT} cell line rendered a stimulation of the transcriptional activity in the majority of the experiments performed; being a particular case which is not seen in other gold standard cell line in the ovarian carcinoma research, OvCar3.

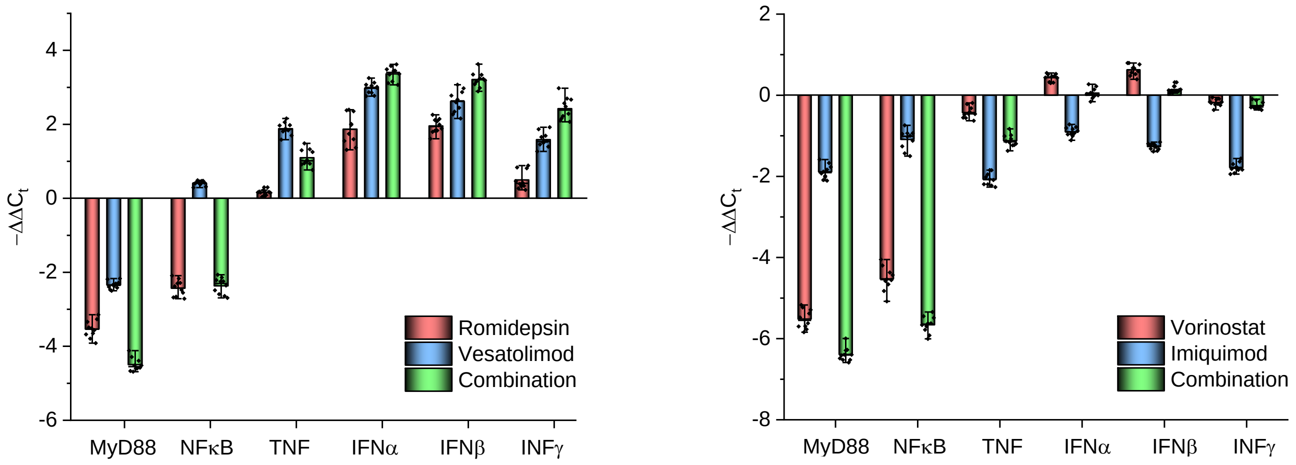

Supplementary Figure 18: PBMCs from 10 healthy volunteers were treated *ex vivo* with 1x IC_{50} for each drug (vorinostat/imiquimod or romidepsin/vesatolimod) for 24 hours. Various mediators of the inflammasome at the transcriptional level were measured by quantitative PCR. Quantification of the potential amplification or reduction of transcription of inflammatory factors by drugs via qPCR is performed using 18S ribosomal RNA as control. The term ΔCt reflects the difference (Δ) in cycle (C) thresholds (t). Differences in ΔCt between treated and untreated cells were shown as ΔΔCt. The results showed downregulation of MyD88 and NFκβ in PBMCs from healthy volunteers treated with vorinostat/imiquimod or romidepsin/vesatolimod. The rest of the genes revealed dissimilar results inherent to their mechanism of actions. Results are representative of n=10 experiments.

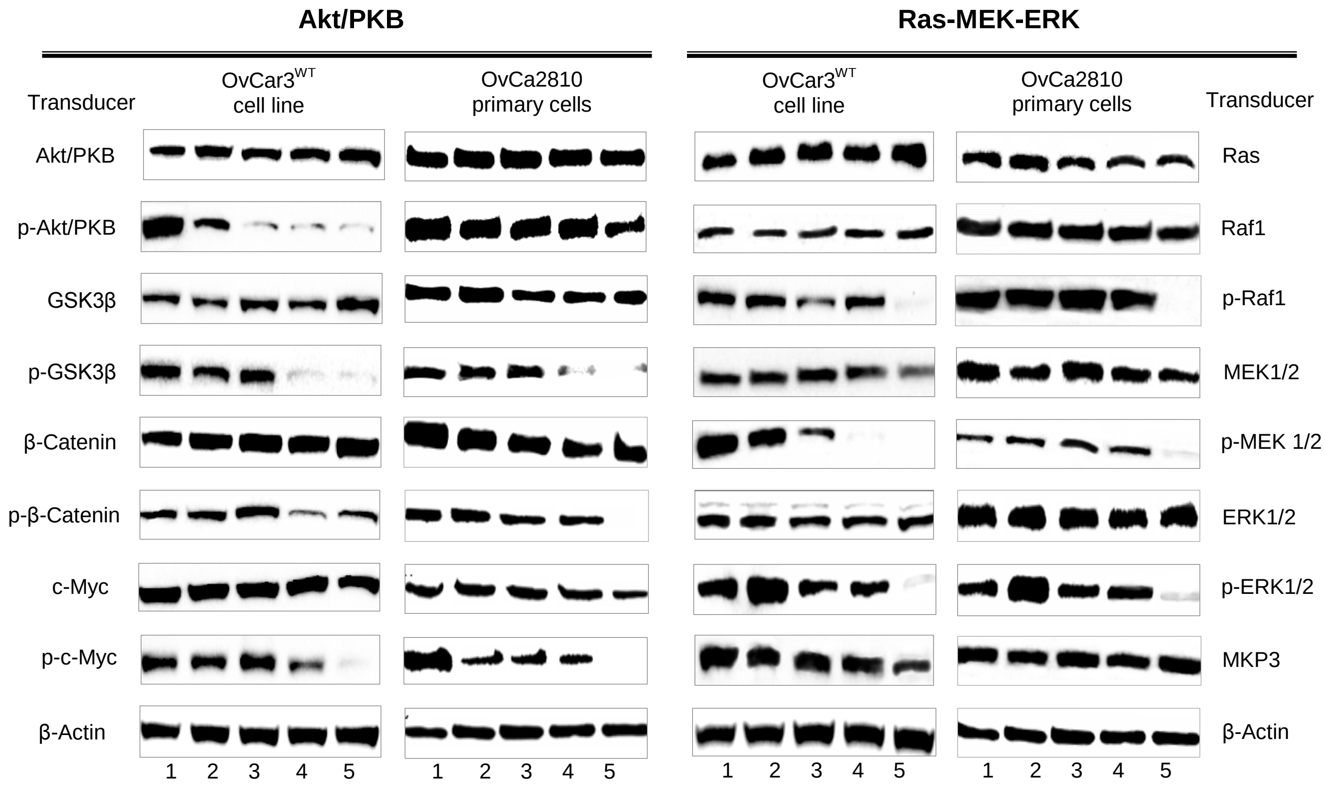

Lanes: **1**: Untreated cells; **2**: DMSO control; **3**: Romidepsin; **4**: Vesatolimod **5**: Romidepsin + Vesatolimod

Supplementary Figure 19: Akt/PKB and Ras-MEK-ERK signal transduction pathways are interrupted by the combination of romidepsin and vesatolimod in OvCar3^{WT} and OvCa2810 cells. In both cascades, it is observed that for many transducers, the protein synthesis was affected by the exposure of single drugs and their combinations. Globally, the phosphate exchange of these transducers was interrupted. Results are representative of n≥3 experiments.

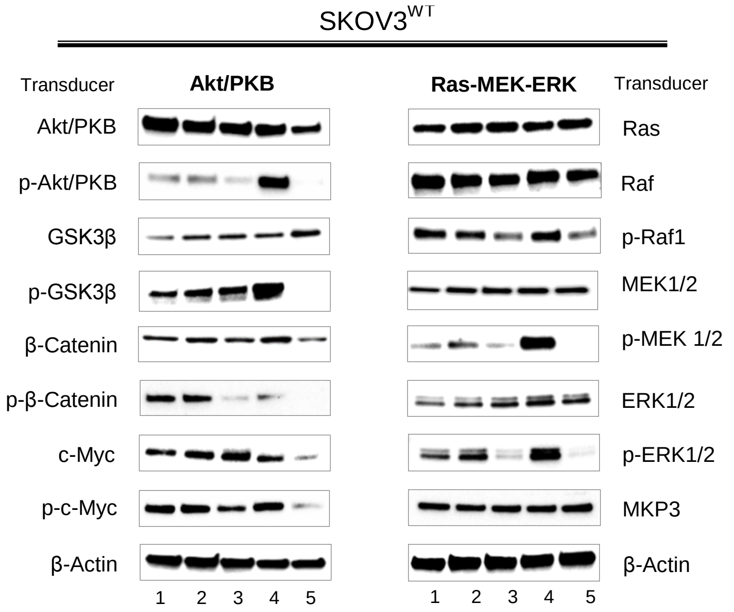

Lanes: **1**: Untreated cells; **2**: DMSO control; **3**: Vorinostat; **4**: Imiquimod **5**: Vorinostat+Imiquimod

Supplementary Figure 20: Akt/PKB and Ras-MEK-ERK signal transduction pathways are interrupted by the combination of vorinostat and imiquimod in SKOV3^{WT} cells. In both cascades, the protein synthesis was affected by the exposure of single drugs and their combinations for many transducers. Globally, the phosphate exchange of these transducers was interrupted. Results are representative of n≥3 experiments.

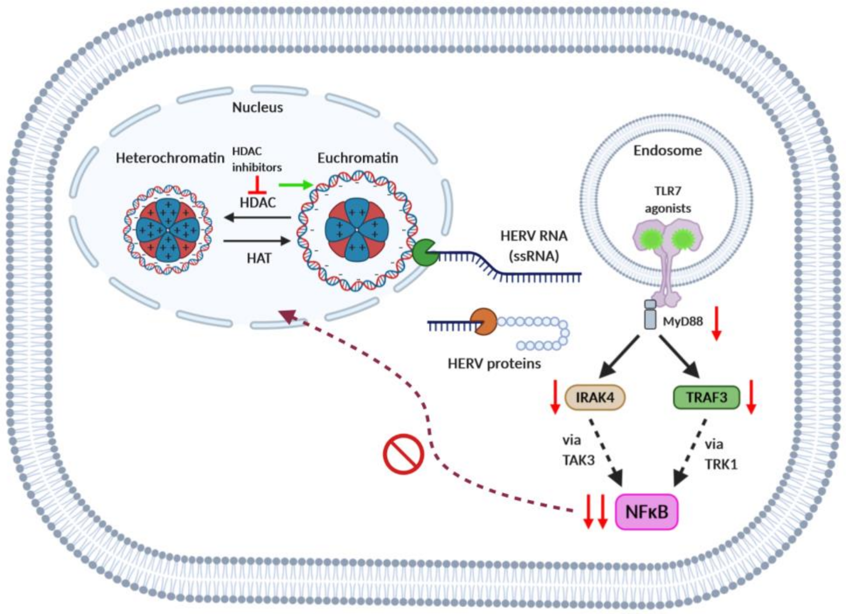

Supplementary Figure 21: Cartoon representing major mechanistic events in “shock and kill” extrapolated to cancer. “Shock and kill” (SaK) is now the dominant strategy intended to eliminate HIV-infected cells in which proviruses are confined to latency by the pressure of antiretroviral therapy (ART). Once infected cells enter latency, viral particles are no longer produced and therefore viral proteins cannot be externally exposed in cell membranes or MHC I. Thus, the immune system is not able to recognize an infected cell. This condition can be chemically reverted by enforcing cells to produce viral RNA and its consequent translation (shock phase). The substances intended to reactivate proviruses artificially are called Latency Reverting Agents (LRA). There is a plethora of compounds which can act as LRAs, among them Histone Deacetylase HDAC inhibitors (HDACis) have been efficiently tested in different *in vitro* models with promising results, demonstrating an excellent capacity of latency reversion in a context of a global gene expression. Thus, the expression of proviral genes by manipulating the cellular epigenetics comprehends the first phase of this approach. A second phase (kill) is then needed for the elimination of viral antigen bearing cells. Different methodologies have been tested. Primordially, the use of the neutralizing antibodies opened an approach to eliminate targeted cells using cellular immune mechanism of clearance. More elegant studies were directed towards creating T-CAR which specifically targets infected cells presenting viral antigens in the membrane. The last approach has cost disadvantages besides the possible systemic aggression against infected tissues. Nevertheless, SaK strategy has been moved to clinical phases with the hope to reduce or eliminate viral reservoirs and provide infected individuals with a more robust immune system.

The use of the innate immunity to force infected cells to go through the process of induced cell death is typically less studied field in Retrovirology. The use of TLR7/8 agonists which recognize (retro)viral signatures is now moving rapidly as a highly promissory approach. However, the single use of those agents do not cure all infected animals, indicating that improvements are needed. Given that the pool of viral RNA as stimuli for TLR7/8 has to reach a triggering level, the co-administration of LRAs may change the play rules.
