# Supplementary material for: Enhanced antitumoral activity of TLR7 agonists via activation of human endogenous retroviruses by HDAC inhibitors
Source: Commun Biol. 2021 Mar 3;4:276. doi: 10.1038/s42003-021-01800-3 (PMC7930250; doi:10.1038/s42003-021-01800-3)
Supplement: Supplementary file 4 — Reporting Summary [file 42003_2021_1800_MOESM4_ESM.pdf]

naturereseach

Corresponding author(s): Dr. Diaz-Carballo

Last updated by author(s): 2021.01.14

Reporting Summary

Nature Research wishes to improve the reproducibility of the work that we publish. This form provides structure for consistency and transparency in reporting. For further information on Nature Research policies, see Authors & Referees and the Editorial Policy Checklist.

Statistics

For all statistical analyses, confirm that the following items are present in the figure legend, table legend, main text, or Methods section.

n/a	Confirmed	
☐	☒	The exact sample size (*n*) for each experimental group/condition, given as a discrete number and unit of measurement
☐	☒	A statement on whether measurements were taken from distinct samples or whether the same sample was measured repeatedly
☐	☒	The statistical test(s) used AND whether they are one- or two-sided<br>*Only common tests should be described solely by name; describe more complex techniques in the Methods section.*
☒	☐	A description of all covariates tested
☒	☐	A description of any assumptions or corrections, such as tests of normality and adjustment for multiple comparisons
☐	☒	A full description of the statistical parameters including central tendency (e.g. means) or other basic estimates (e.g. regression coefficient) AND variation (e.g. standard deviation) or associated estimates of uncertainty (e.g. confidence intervals)
☐	☒	For null hypothesis testing, the test statistic (e.g. *F*, *t*, *r*) with confidence intervals, effect sizes, degrees of freedom and *P* value noted<br>*Give P values as exact values whenever suitable.*
☒	☐	For Bayesian analysis, information on the choice of priors and Markov chain Monte Carlo settings
☒	☐	For hierarchical and complex designs, identification of the appropriate level for tests and full reporting of outcomes
☒	☐	Estimates of effect sizes (e.g. Cohen's *d*, Pearson's *r*), indicating how they were calculated

Our web collection on statistics for biologists contains articles on many of the points above.

Software and code

Policy information about availability of computer code

Data collection	
Data analysis	CFX Meastro 4.0.2325.0418, Bio-Rad<br>Excel 2010, Microsoft<br>Image Lab 5.2.1, Bio-Rad<br>Origin Pro 2021, 9.8.0.200 Origin Lab Corporation<br>CytExpert 2.2, Beckman Coulter<br>i control 1.6, Tecan<br>Advanced Research 5.11.02, Nikon<br>Basic Research 4.13.04, Nikon

For manuscripts utilizing custom algorithms or software that are central to the research but not yet described in published literature, software must be made available to editors/reviewers. We strongly encourage code deposition in a community repository (e.g. GitHub). See the Nature Research guidelines for submitting code & software for further information.

Data

Policy information about availability of data

All manuscripts must include a data availability statement. This statement should provide the following information, where applicable:

- Accession codes, unique identifiers, or web links for publicly available datasets
- A list of figures that have associated raw data
- A description of any restrictions on data availability

All data will be provided for further analysis in case of neccessity

Field-specific reporting

Please select the one below that is the best fit for your research. If you are not sure, read the appropriate sections before making your selection.

☒ Life sciences ☐ Behavioural & social sciences ☐ Ecological, evolutionary & environmental sciences

For a reference copy of the document with all sections, see nature.com/documents/nr-reporting-summary-flat.pdf

Life sciences study design

All studies must disclose on these points even when the disclosure is negative.

Sample size	For animal studies, we employed 4 isogenic animals per group and repeated the experiment 4 times
Data exclusions	We did not exclude any data
Replication	All experiments were performed at least 4 times independently and the results were replicated.
Randomization	We have randomized animals in the xenograft studies
Blinding	*Describe whether the investigators were blinded to group allocation during data collection and/or analysis. If blinding was not possible, describe why OR explain why blinding was not relevant to your study.*

Reporting for specific materials, systems and methods

We require information from authors about some types of materials, experimental systems and methods used in many studies. Here, indicate whether each material, system or method listed is relevant to your study. If you are not sure if a list item applies to your research, read the appropriate section before selecting a response.

Materials & experimental systems

n/a	Involved in the study	
☐	☒	Antibodies
☐	☒	Eukaryotic cell lines
☒	☐	Palaeontology
☐	☒	Animals and other organisms
☐	☒	Human research participants
☒	☐	Clinical data

Methods

n/a	Involved in the study	
☐	☒	ChIP-seq
☐	☒	Flow cytometry
☒	☐	MRI-based neuroimaging

Antibodies

Antibodies used

Caspase 9 , Cell Signaling Technology , Catalog # 9502S , Clone: (-) , Lot # 8
cleaved Caspase 9 , Cell Signaling Technology , Catalog # 20750S , Clone: Asp315, D819E , Lot # 1
Caspase 3 , Cell Signaling Technology , Catalog # 9662S , Clone: (-) , Lot # 18
cleaved Caspase 3 , Cell Signaling Technology , Catalog # 9664S , Clone: D175, 5A1E , Lot # 21
PARP , Cell Signaling Technology , Catalog # 9532S , Clone: 46D11 , Lot # 9
cleaved PARP , Cell Signaling Technology , Catalog # 5626S , Clone: D64I10 , Lot # 30
ERK 1/2 (MAPK) , Cell Signaling Technology , Catalog # 4695S , Clone: 137F5 , Lot # 28
pERK 1/2 (pMAK) , Cell Signaling Technology , Catalog # 4370S , Clone: T202/Y204, D13.14.4E , Lot # 24
MEK 1/2 , Cell Signaling Technology , Catalog # 4694S , Clone: L38C12 , Lot # 2
pMEK 1/2 , Cell Signaling Technology , Catalog # 9121S , Clone: S217/221 , Lot # 31
MyD88 , Cell Signaling Technology , Catalog # 4283 , Clone: D80F5 , Lot # 25
Akt-PKB , Cell Signaling Technology , Catalog # 9272S , Clone: (-) , Lot # 28
pAkt , Cell Signaling Technology , Catalog # 4060S , Clone: S473, D9E , Lot # 25
β-Catenin , Cell Signaling Technology , Catalog # 9582S , Clone: 6B3 , Lot # 5

p-β-Catenin , Cell Signaling Technology , Catalog # 9561T , Clone: S33/37/T41 , Lot # 30
GSK3-β , Cell Signaling Technology , Catalog # 9315S , Clone: 27C 10 , Lot # 14
c-Myc , Cell Signaling Technology , Catalog # 5605S , Clone: D84C12 , Lot # 15
p-c-Myc , Cell Signaling Technology , Catalog # 9401S , Clone: Thr58/Ser62 , Lot # 2
NF-κB p65 , Cell Signaling Technology , Catalog # 8242S , Clone: D14E12, XP(r) , Lot # 9
Ras , Cell Signaling Technology , Catalog # 3339S , Clone: 27H5 , Lot # 3
BcLxL , Cell Signaling Technology , Catalog # 2764S , Clone: 54H6 , Lot # 6
MKP3 , Elabscience , Catalog # E-AB-32042 , Clone: (-) , Lot # DK7219
c-Raf , Cell Signaling Technology , Catalog # 9422S , Clone: (-) , Lot # 3
Vimentin , Invitrogen , Catalog # MA5-11883 , Clone: V9 , Lot # SE2389382
p-c-Raf , Cell Signaling Technology , Catalog # 9431S , Clone: S289/296/301 , Lot # 2
β-Tubulin , Santa Cruz Biotechnology , Catalog # sc-55529 , Clone: G-8 , Lot # C2713
β-Actin-Peroxidase , Sigma Aldrich , Catalog # A3854 , Clone: AC15 , Lot # 048M4859V

HERV-V1/V2 is custom designed and produced NovoPro Bioscience

H3acK9 , Cell Signaling Technology , Catalog # 9649 , Clone: D1H2 , Lot # 9
H3acK14 , Cell Signaling Technology , Catalog # 7627 , Clone: D4B9 , Lot # 5
H3acK18 , Cell Signaling Technology , Catalog # 13998 , Clone: D8Z5H , Lot # 1
H3acK27 , Cell Signaling Technology , Catalog # 8173 , Clone: D5E4 , Lot # 4
NF-κB p105/p50 , Cell Signaling Technology , Catalog # 13586 , Clone: D4P4D , Lot # 2
IKBα , Biolegend , Catalog # 662402 , Clone: 3D6C02 , Lot # B291366
pIKBα (S32) , Cell Signaling Technology , Catalog # 2859 , Clone: 14D4 , Lot # 18

Syncytin1/HERV WE1 , biorbyt , Catalog # orb124303 , Clone: polyclonal , Lot # A2908
Syncytin2/HERV FRD1 , biorbyt , Catalog # orb157507 , Clone: polyclonal , Lot # E11167
HERV MER34-1 , Atlas Antibodies , Catalog # HPA011423 , Clone: polyclonal , Lot # R03099
HERV 3.1 , biorbyt , Catalog # orb31513 , Clone: polyclonal , Lot # E0395

PE mouse anti cleaved PARP, BD Pharmingen, Catalog # 552933, Clone: Asp214, Lot # 9165966
PE mouse IgG1, Kappa isotype control, BioLegend, Catalog# 400113, Clone: MOPC-21, Lot# B244595
Anti-Rabbit IgG HRP-linked Antibody , Cell Signaling Technology , Catalog # 7074S , Clone: (-) , Lot # 28
Anti-Mouse IgG HRP-linked Antibody , Cell Signaling Technology , Catalog # 7076S , Clone: (-) , Lot # 33
Anti-mouse IgG Fab2 Alexa Fluor ® 488 , Cell Signaling Technology , Catalog # 4408S , Clone: (-) , Lot # 18
Anti-mouse IgG Fab2 Alexa Fluor ® 555 , Cell Signaling Technology , Catalog # 4409S , Clone: (-) , Lot # 14
Anti-rabbit IgG Fab2 Alexa Fluor ® 488 , Cell Signaling Technology , Catalog # 4412S , Clone: (-) , Lot # 20
Anti-rabbit IgG Fab2 Alexa Fluor ® 555 , Cell Signaling Technology , Catalog # 4413S , Clone: (-) , Lot # 16

Validation

Caspase 9 - WB, FACS validated
cleaved Caspase 9 - WB, FACS, IF validated
Caspase 3 - WB, IHC validated
cleaved Caspase 3 - WB, FACS, IF, IHC validated
PARP - WB, FACS, IF validated
cleaved PARP - WB, FACS, IF, IHC validated
ERK 1/2 (MAPK) - WB, FACS, IF, IHC validated
pERK 1/2 (pMAK) - WB, FACS, IF, IHC validated
MEK 1/2 - WB validated
pMEK 1/2 - WB validated
MyD88 - WB validated
Akt-PKB - WB, FACS, IF validated
pAkt - WB, FACS, IF, IHC validated
β-Catenin - WB, IHC validated
p-β-Catenin - WB validated
GSK3-β - WB, IHC validated
c-Myc - WB, FACS, IF validated
p-c-Myc - WB validated
NF-κB p65 - WB, FACS, IF, IHC validated
Ras - WB Validated
BcLxL - WB, FACS, IHC validated
MKP3 - WB validated
c-Raf - WB validated
Vimentin - IF validated
p-c-Raf - WB validated
β-Tubulin - WB, IF, IHC validated
β-Actin-Peroxidase - WB validated
PE mouse anti cleaved PARP - FACS validated
HERV-V1/V2 - WB, FACS, IF, IHC validated in our laboratory
H3acK9 - WB validated
H3acK14 - WB validated
H3acK18 - WB validated
H3acK27 - WB validated
NF-κB p105/p50 - WB validated
IKBα - WB validated
pIKBα (S32) - WB validated

Syncytin1/HERV WE1 - WB validated
Syncytin2/HERV FRD1 - WB validated
HERV MER34-1 - WB validated
HERV 3.1 - WB validated

Eukaryotic cell lines

Policy information about cell lines

Cell line source(s)	Institute of Cell Biology, University Hospital Essen
Authentication	None of the cell lines used were authenticated
Mycoplasma contamination	All cell lines were tested negative for mycoplasma using the MycoSPY PCR kit from Biontex
Commonly misidentified lines (See ICLAC register)	*Name any commonly misidentified cell lines used in the study and provide a rationale for their use.*

Animals and other organisms

Policy information about studies involving animals; ARRIVE guidelines recommended for reporting animal research

Laboratory animals	NMRI nude mice, Female, six to eight week old
Wild animals	The study did not involve wild animals
Field-collected samples	The study did not involve samples collected from the field
Ethics oversight	Ethics Commission of the Ruhr-University of Bochum (register numbers: 4042-11 and 5235-15)

Note that full information on the approval of the study protocol must also be provided in the manuscript.

Human research participants

Policy information about studies involving human research participants

Population characteristics	Ascites was collected from ten ovarian carcinoma patients. Age 45 to 65 years.<br>Blood was collected from 10 male and 10 female healthy volunteers for isolation of PBMCs. Age 25 to 40 years.<br>Samples were fully anonymized and no genetic studies were performed.
Recruitment	*Describe how participants were recruited. Outline any potential self-selection bias or other biases that may be present and how these are likely to impact results.*
Ethics oversight	Ethics Commission of the Ruhr-University of Bochum (register numbers: 4042-11 and 5235-15)

Note that full information on the approval of the study protocol must also be provided in the manuscript.

ChIP-seq

Data deposition

- [ ] Confirm that both raw and final processed data have been deposited in a public database such as GEO.
- [x] Confirm that you have deposited or provided access to graph files (e.g. BED files) for the called peaks.

Data access links<br>*May remain private before publication.*	Gene Expression Omnibus, Accession number GSE164809
Files in database submission	B_peaks_UNTREATED.bed<br>D_peaks_VORINOSTAT1.bed<br>D_peaks_VORINOSTAT2.bed<br>F_peaks_ROMIDEPSIN1.bed<br>F_peaks_ROMIDEPSIN2.bed
Genome browser session (e.g. UCSC)	https://genome.ucsc.edu/index.html

Methodology

Replicates	6 samples (1x untreated, 1x vorinostat, 1x romidepsin plus 3 corresponding DNA input samples) During ChIP seq the

Replicates	vorinostat and romidepsin samples were split into two.
Sequencing depth	averagely generating 54370600 raw reads and 54201400 clean reads; Average read depth was 2700000000; a least 94.9% of the reads were mapped.
Antibodies	H3acK9, Cell Signaling Technology , Catalog # 9649 , Clone: D1H2 , Lot # 9
Peak calling parameters	Only the alignments within 2 mismatches were considered in Peak calling. MACS (Model-based Analysis for ChIP -Seq, version: MACS-1.4.2) was used then for Peak calling. Parameters for Peak calling are: macs14 -s 49 -g hs -p 1e-5 -w --space 50 -m 10,30
Data quality	Data filtering parameters: Clean Parameter: SOAPnuke filter -l 5 -q 0.5 -n 0.1 -Q 2 -c 50<br><br>Data filtering includes removing adaptor sequences, contamination and low-quality reads from raw reads. These reads were detected by BGI programs. Low-quality reads include three types, and the read which could be accord with anyone of the three types will be removed:<br>1) The ratio of N in whole read was over 10%;<br>2) Remove reads in which unknown bases are more than 10%;<br>3) The ratio of base whose quality was less than 20 was over 10%.
Software	Data filtering: SOAPnuke,<br>Data alignment: SOAPaligner/SOAP2/BWA,<br>Different peak between samples: MAnorm<br>Peak calling: MACS

Flow Cytometry

Plots

Confirm that:

☐ The axis labels state the marker and fluorochrome used (e.g. CD4-FITC).

☒ The axis scales are clearly visible. Include numbers along axes only for bottom left plot of group (a 'group' is an analysis of identical markers).

☒ All plots are contour plots with outliers or pseudocolor plots.

☒ A numerical value for number of cells or percentage (with statistics) is provided.

Methodology

Sample preparation	Cells were harvested and fixed with methanol/acetone (1:1) and blocked with 5% BSA in PBST for 30 min.<br>Conjugated antibodies were incubated according to manufacturer's recommendations in 1% BSA in PBST for 20 min.<br>Unconjugated primary antibodies were incubated 1:100 in 1 % BSA in PBST for 2 hours, after that the cells were incubated with the secondary antibody 1:1000 in 1 % BSA in PBST for 1 hour and measurement was performed. Washing steps with PBST were included between antibody incubations and before measurement.<br><br>All FACS experiments were performed with SKOV3 WT (ATCC® HTB-77™)
Instrument	CytoFLEX, Serial No: 40756525, Beckman Coulter
Software	CytExpert 2.2, Beckman Coulter
Cell population abundance	We did not sort cells.
Gating strategy	The main population of the cells were gated within the FSC/SSC dot plot according to density. Histograms of isotype controls were used to set the gate for the negative signals. Everything within that gate was considered as a positive signal.

☐ Tick this box to confirm that a figure exemplifying the gating strategy is provided in the Supplementary Information.
