# Supplementary material for: Enhanced antitumoral activity of TLR7 agonists via activation of human endogenous retroviruses by HDAC inhibitors
Source: Commun Biol. 2021 Mar 3;4:276. doi: 10.1038/s42003-021-01800-3 (PMC7930250; doi:10.1038/s42003-021-01800-3)
Supplement: Supplementary file 6 — Description of Additional Supplementary Files [file 42003_2021_1800_MOESM6_ESM.pdf]

Description of Additional Supplementary Files

File name: Supplementary Data 1

Description: Overview of the HERV-loci differentially expressed in $SKOV3^{WT}$ ovarian carcinoma cells treated with the HDACis romidepsin and vorinostat. Diffpeaks extracted from H3AcK9 chromatin immunoprecipitation. ChIP-enriched DNA sequence reads were mapped to reference genome (hg38) data base. Data included the Log_{10} p-values diffpeaks of two biological replicates.

File name: Supplementary Data 2

Description: Raw data for all main figures from the article.
